# When readiness fails: a TOE–TAM mixed-methods analysis of AI-enabled HR digitalization in a resource-constrained small island context

**DOI:** 10.3389/frai.2026.1902314

**Published:** 2026-09-21

**Authors:** Jecha S. Jecha, Ally Khamis Ali, Adilu Mussa

**Affiliations:** 1 Faculty of Science, Zanzibar University, Zanzibar, Tanzania; 2 Faculty of Arts and Social Sciences, Department of Public Administration. Zanzibar University, Zanzibar, Tanzania; 3 Faculty of Business Administration, Department of Accounting and Finance. Zanzibar University, Zanzibar, Tanzania

**Keywords:** AI adoption, human resource management, institutional decoupling, small island contexts, technology sovereignty, TOE–TAM

## Abstract

This study examines barriers to adopting artificial intelligence (AI) in human resource management (HRM)—hereafter AI-enabled human resources (AI-HR)—across Zanzibar’s public and private sectors and asks whether the standard predictors of an integrated Technology–Organization–Environment and Technology Acceptance Model (TOE–TAM) framework behave as theorized under mandatory, resource-constrained conditions. An explanatory sequential mixed-methods design combined a survey of HR professionals, information technology (IT) managers, and administrators (*N* = 137), analyzed with partial least squares structural equation modelling (PLS-SEM), with nine semi-structured key-informant interviews used to explain the quantitative results. The organizational readiness (OR) scale failed internal consistency (Cronbach’s α = −0.105) and was therefore excluded before structural testing. Of the six structurally interpretable paths, perceived ease of use strongly predicted perceived usefulness (*β* = 0.552, *p* < 0.001), while technological readiness predicted AI-HR adoption readiness only marginally (*β* = 0.216, *p* = 0.044; bias-corrected and accelerated [BCa] confidence interval that included zero). Environmental readiness, perceived usefulness, and perceived ease of use did not predict adoption readiness, and adoption readiness did not predict HRM effectiveness. The interviews identified five mechanisms omitted from the reduced model: stalled, partial digitalization; infrastructure and vendor dependence as a binding constraint; capacity deficits and expertise flight; institutional decoupling between formal policy and operational practice; and a cultural preference for human judgement under directive adoption. The findings suggest that AI-HR adoption in resource-constrained small island contexts depends on foundational infrastructure, institutional coherence, and technology sovereignty, while also motivating a sequenced policy agenda and more context-sensitive measurement.

## 1 Introduction

Human resource management is undergoing a structural shift. The function that once centered on administrative processing is increasingly expected to operate strategically, and that strategic turn is being mediated by artificial intelligence (AI). AI-enabled HR systems are promoted on the basis of efficiency gains and richer workforce analytics, yet their deployment encounters a stack of mutually reinforcing institutional obstacles. Where organizations lack standardized frameworks for governing AI-related risk, they struggle to triage those risks or to sustain ethical oversight of HR practice (Hall, 2026). Evidence on digital transformation leadership reinforces the point: aligning technological change with organizational capability depends on relational work and operational innovation rather than on technology procurement alone (Van Roekel et al., 2025).

The bulk of this evidence originates in private-sector firms located in Global North economies that already possess functioning regulatory regimes and dependable digital infrastructure. Public administrations operate under a different incentive structure, weighting citizen welfare and procedural fairness above innovation for its own sake (Schiff et al., 2021). The result is a distinct set of governance demands and a slower adoption trajectory, a divergence that widens in resource-constrained settings. Small Island Developing States (SIDS) confront an additional layer of structural constraint: narrow fiscal space, immature information and communication technology (ICT) infrastructure, and reliance on external donor financing, each of which complicates any digital transformation agenda (Faustine and Rachmawati, 2024; Mwita and Kitole, 2025; Venugopal et al., 2024).

Zanzibar exemplifies these tensions. It is a semi-autonomous archipelago within the United Republic of Tanzania and is not separately included on the United Nations list of Small Island Developing States (United Nations Office of the High Representative for the Least Developed Countries, Landlocked Developing Countries and Small Island Developing States, n.d.). The study therefore treats Zanzibar as a resource-constrained small island context, not as a formally designated SIDS. The analytical comparison remains relevant because Zanzibar faces several conditions widely discussed in the SIDS literature, including narrow fiscal space, infrastructure constraints, donor dependence, and limited institutional capacity. It must build connectivity, data architecture, and computing capacity alongside regulation, standards, and workforce capability, a dual burden that can outstrip available policymaking capacity. Most developing economies lack the institutional scaffolding for AI governance that established regulatory blocs take for granted (Nahar, 2026). AI integration in HR also carries documented risks, including algorithmic bias, privacy exposure, diminished collaboration, and job reconfiguration (Wójcik, 2025), which threatens performance and employee trust where safeguards are thin.

Three gaps follow. First, evidence on how organizational and environmental readiness condition AI adoption across public and private organizations in resource-constrained small island and Global South settings remains thin (Isagah and Dhaou, 2024). Second, the joint influence of institutional constraints, governance pressures, and individual perceptions on adoption readiness and HRM effectiveness under resource-constrained conditions remains poorly specified. Third, these omissions limit the transferability of established adoption frameworks to small, donor-dependent economies such as Zanzibar, where assumed background conditions frequently do not hold.

Against this backdrop, the study asks how technological, organizational, and environmental readiness shape AI adoption readiness and HRM effectiveness across Zanzibar’s public and private sectors, and whether the predictors of an integrated TOE–TAM model behave as theorized under mandatory, resource-constrained conditions. The hypothesized framework contained Technological Readiness (TR), Organizational Readiness (OR), Environmental Readiness (ER), Perceived Usefulness (PU), Perceived Ease of Use (PEOU), AI Adoption Readiness (AIR), and HRM Effectiveness (HRME) (Islam et al., 2023; Qu and Kim, 2025). OR failed the measurement assessment and was excluded before structural testing. Its failure is retained as a standalone measurement finding and interpreted alongside the interview evidence on institutional decoupling. The qualitative phase then explains the reduced model’s predominantly null findings through the accounts of those responsible for digital transformation in Zanzibar’s institutions.

## 2 Literature review and conceptual framework

### 2.1 Integrating the TOE (macro) and TAM (micro) lenses

The Technology–Organization–Environment (TOE) framework accounts for organizational technology adoption across three contexts: technological conditions, organizational resources and structure, and the external environment of industry, policy, and regulation (Tornatzky and Fleischer, 1990). In Tornatzky and Fleischer’s (1990) formulation, adoption decisions register both internal capability and external constraint.

The Technology Acceptance Model (TAM) works at the level of the individual. It holds that acceptance of a system turns on two perceptions: perceived usefulness (PU), the belief that the system improves job performance, and perceived ease of use (PEOU), the belief that using it demands little cognitive effort (Davis, 1989). Across diverse systems, PU and PEOU predict intentions to use, and PEOU operates as an antecedent of PU (Venkatesh and Davis, 2000).

The two lenses are complementary in Zanzibar’s public and private sectors. TOE captures the macro-level conditions: infrastructure, organizational resources, and policy within which adoption is even possible, while TAM captures the micro-level attitudes of the HR officers who operate the tools. Studies that integrate the two confirm that contextual and psychological factors jointly govern AI use in HR (Chatterjee et al., 2021). In the Tanzanian case, Faustine and Rachmawati (2024) show that technological, organizational, and environmental factors together determine whether enterprises take up AI (Faustine and Rachmawati, 2024). The implication is that AI-HR adoption is bounded by infrastructure and policy at the TOE level, while its success at the individual level depends on HR professionals finding the tools both useful and manageable.

### 2.2 Technological context: infrastructure as a binding constraint (H1)

The technological context comprises the technologies in use and available for use: ICT infrastructure, system reliability, integration capacity, and cybersecurity. Weak connectivity, unstable power, legacy systems, and inadequate security are documented impediments to AI adoption in developing economies (Sadat et al., 2024). Faustine and Rachmawati (2024) report that technological factors drive both AI adoption and HRM effectiveness among Tanzanian enterprises (Faustine and Rachmawati, 2024), and evidence from Ghana shows that e-HRM tools improved HR systems only where the underlying infrastructure was already in place (Asamoah-Appiah and Kesari, 2023). Because infrastructure in Zanzibar is uneven, technological readiness is expected to act as a binding constraint.

*H1*: Technological readiness positively affects AI-enabled HR adoption readiness in organizations in Zanzibar.

### 2.3 Organizational context: budget, culture, and management support (H2)

The organizational context encompasses firm size and structure, financial resources, HR capabilities, culture, and managerial commitment. Whether an AI project is funded, aligned with strategy, and embedded in core HR workflows depends substantially on management support and organizational readiness (Islam et al., 2023). Hierarchical cultures and limited digital skills can obstruct e-HRM implementation even when the requisite technologies are present (Muzaffar et al., 2024).

*H2*: Organizational readiness positively affects AI-enabled HR adoption readiness in organizations in Zanzibar.

### 2.4 Environmental context: policy and regulatory pressure (H3)

The environmental context refers to conditions outside the organization: government policy, regulation, and competitive pressure. In developing countries, governments steer ICT and AI adoption through digital strategies and data-protection regimes (Shahadat et al., 2023), and coherent digital policy accelerates e-government uptake, whereas fragmented policy is associated with project failure (Asamoah-Appiah and Kesari, 2023). Because AI-HR in Zanzibar is bound up with the broader digital-economy agenda, environmental readiness is expected to generate institutional pressure for adoption.

*H3*: Environmental readiness (including government policies and competitive pressures) positively affects AI-enabled HR adoption readiness in organizations in Zanzibar.

### 2.5 TAM: user perceptions of AI-HR tools (H4, H5, H6)

TAM holds that perceived usefulness and perceived ease of use shape attitudes, intentions, and use (Mohd Amir et al., 2020). Davis (1989) established that PEOU feeds into PU, a relationship replicated across contexts (Venkatesh and Davis, 2000). In AI-enabled HRM research, professionals’ perceptions of usefulness and ease of use predict their intention to adopt AI tools (Xing et al., 2025), and both perceptions were significant alongside TOE factors in explaining AI adoption in HR (Islam et al., 2023). Given that Zanzibar’s HR officers vary in digital skill and in language preference, PU and PEOU are expected to shape uptake once deployment is feasible.

*H4*: Perceived ease of use of AI-enabled HR tools positively affects perceived usefulness.

*H5*: The perceived usefulness of AI-enabled HR tools positively affects AI-enabled HR adoption readiness.

*H6*: Perceived ease of use of AI-enabled HR tools positively affects AI-enabled HR adoption readiness.

### 2.6 From adoption readiness to HRM effectiveness (H7)

Properly implemented e-HRM tools improve HRM effectiveness and organizational performance (Agarwal, 2022). The Ghanaian evidence shows e-HRM enhancing HRM effectiveness with downstream organizational consequences (Asamoah-Appiah and Kesari, 2023), and Faustine and Rachmawati (2024) report a positive effect of AI adoption on HRM effectiveness in Tanzanian enterprises. Here, AIR denotes an organization’s intent and capability to implement AI in HR, and HRME denotes improvements in HR service quality, data reliability, and strategic contribution.

*H7*: AI-enabled HR adoption readiness positively affects perceived HRM effectiveness in organizations in Zanzibar.

### 2.7 Conceptual framework

The conceptual model proposes that technological, organizational, and environmental readiness shape AI adoption readiness, while perceived usefulness and perceived ease of use influence AI adoption readiness directly and through the classical TAM pathway; adoption readiness is then expected to raise HRM effectiveness (Faustine and Rachmawati, 2024; Asamoah-Appiah and Kesari, 2023). Figure 1 presents the seven hypotheses specified before the measurement assessment.

**Figure 1:**
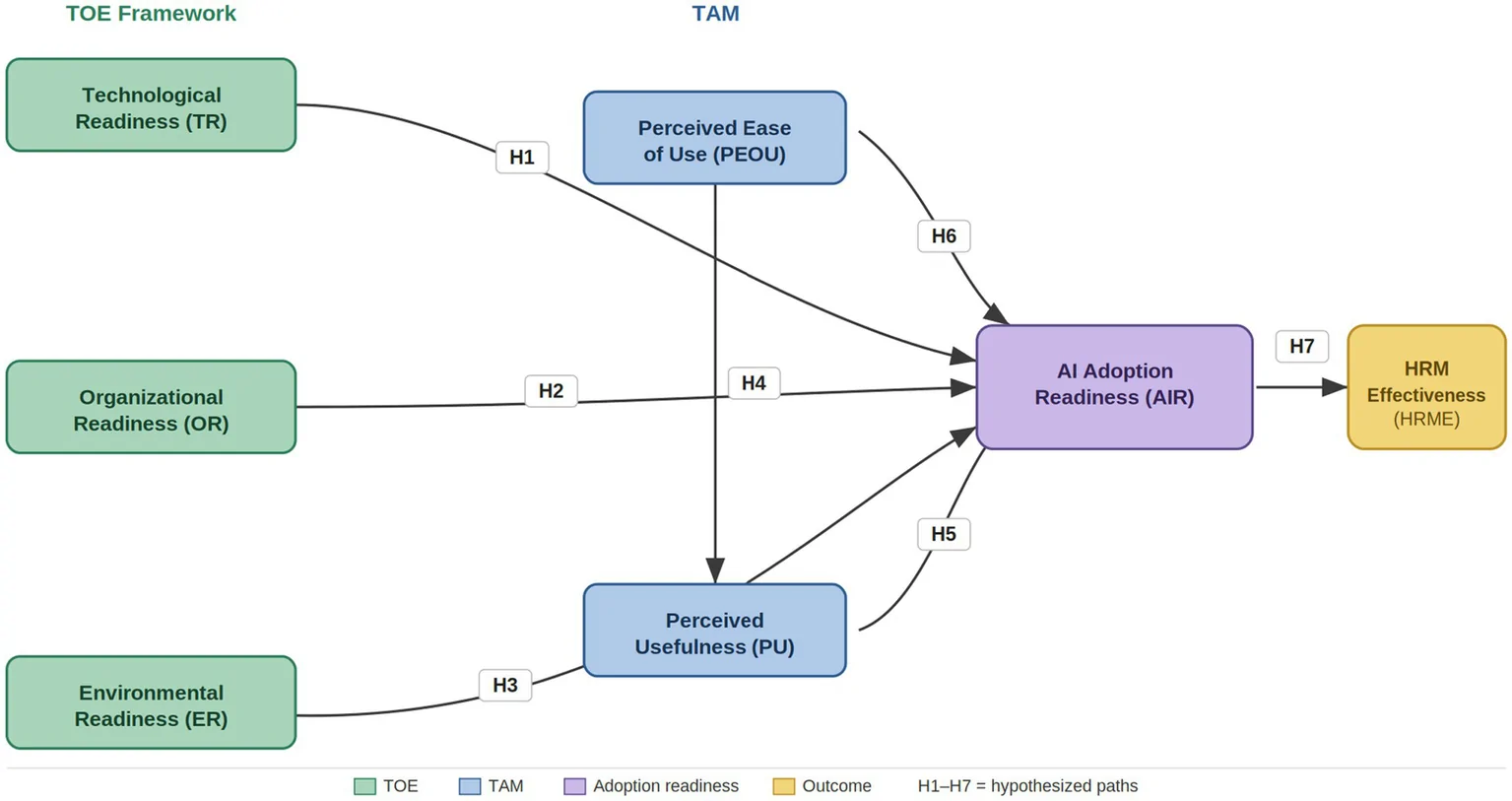
Hypothesized conceptual framework integrating the TOE and TAM perspectives. H1–H7 denote the hypothesized paths tested in the study.

## 3 Method

### 3.1 Research design

The study used an explanatory sequential mixed-methods design, in which a quantitative phase precedes and informs a qualitative phase whose purpose is to explain the quantitative results (Creswell, 2018; Ivankova et al., 2005). The phases are joined by an explicit rationale: the qualitative phase exists because the survey produced results that a survey alone cannot account for. In Phase 1, a structured survey (*N* = 137) was analyzed with PLS-SEM to estimate the integrated TOE–TAM model. In Phase 2, nine semi-structured key-informant interviews were conducted to explain the predominantly null findings. Integration occurred at three points: the quantitative results defined what required explanation; the interview informants were selected for their capacity to speak to the non-significant paths; and the qualitative material is embedded in the hypothesis-by-hypothesis discussion.

### 3.2 Research context and sampling

#### 3.2.1 Quantitative phase

Zanzibar is a semi-autonomous small island jurisdiction within Tanzania; it is not separately designated as one of the SIDS by the United Nations (United Nations Office of the High Representative for the Least Developed Countries, Landlocked Developing Countries and Small Island Developing States, n.d.). The context nevertheless shares several vulnerabilities discussed in the small-island development literature, including constrained ICT infrastructure and reliance on external financing for technology initiatives (Faustine and Rachmawati, 2024; Mwita and Kitole, 2025). The target population comprised HR professionals, IT managers, and administrators in public and private institutions engaged in the transition to digital HR workflows. The achieved sample (*N* = 137) comprised 86 respondents from public-sector or government agencies (62.8%), 39 from the private sector (28.5%), 6 from small and medium-sized enterprises (SMEs) (4.4%), 5 from non-governmental organizations (NGOs) (3.7%) or civil society organizations (3.6%), and 1 reporting a combined private-sector/SME affiliation (0.7%). By role, the sample included 57 HR managers and staff (41.6%), 56 in other roles (40.9%), 14 in general management (10.2%), and 10 IT managers and staff (7.3%). Professional experience ranged from 1 to 53 years (median [Mdn] = 5).

No sampling frame exists for the target population, because Zanzibar maintains no register of HR, IT, and administrative staff engaged in digital HR work. Probability sampling was therefore not feasible, and a non-probability strategy was used: organizations known to be undertaking HR digitalization were approached through institutional gatekeepers, eligible staff within them were invited purposively by role, and respondents were asked to refer comparable colleagues. Participation was voluntary and anonymous. The achieved size was evaluated using the inverse square root method for PLS-SEM, which estimates the minimum sample required to detect a given path at 5% significance and 80% power (Kock and Hadaya, 2018). At *N* = 137, the smallest detectable standardized coefficient is approximately 0.21. The sample is therefore adequate for effects at or above that magnitude, including the two paths that reached significance, but it is underpowered for the small effects observed elsewhere in the model (*β* ≤ 0.14). Several null results are consequently statistically inconclusive rather than evidence of no relationship, and they are interpreted accordingly throughout Sections 5 and 6.

Three sources of sampling bias follow and constrain the claims the study can support. First, the sample is weighted towards the public sector (62.8%), which reflects the dominance of government and parastatal bodies in Zanzibar’s formal employment and in current digitalization initiatives, but it means the findings speak primarily to public and large formal organizations. Second, SMEs (4.4%) and IT specialists (7.3%) are thinly represented, so the model cannot characterize adoption dynamics in small private firms, and the technological readiness construct rests largely on the assessments of non-specialist respondents. Third, recruitment through gatekeepers and referral invites self-selection among organizations and individuals already engaged with digitalization, which would tend to overstate rather than understate readiness. That direction matters for interpretation: a sample skewed towards the more digitally engaged still produced weak and largely null structural relations, which makes the central finding conservative rather than an artefact of who responded.

#### 3.2.2 Qualitative phase

Nine key informants took part in semi-structured interviews of 45–60 min. They comprised HR directors and senior HR officers, an ICT services coordinator, a finance director, and a machine-learning specialist drawn from university, government, and private organizations. Informants were purposively selected for prior engagement with digital HR systems and their capacity to explain the non-significant quantitative paths, consistent with explanatory sequential designs (Ivankova et al., 2005). A formal saturation threshold was not claimed. Sample adequacy was judged through information power: the study had a focused explanatory aim, highly specific informants, an established TOE–TAM frame, and interviews directed at a defined set of unexpected quantitative results (Malterud et al., 2016). During iterative analysis, adequacy was evaluated based on whether the accounts provided explanatory coverage of those results. The interview guide moved from role and context to TOE factors, perceived value and effectiveness, and policy recommendations.

#### 3.2.3 Unit of analysis and level of measurement

The constructs operate at two levels, and the study is explicit about how they were measured. TOE constructs (technological, organizational, and environmental readiness) describe organizational and environmental conditions, whereas TAM constructs (perceived usefulness and ease of use) describe individual cognition. All seven constructs were measured through individual respondents. For the TOE constructs, respondents acted as key informants reporting perceptions of conditions in their organizations; no scores were aggregated to the organizational level, and no organizational identifier was collected, so the effective unit of analysis throughout is the individual respondent’s perception rather than the organization as an entity. Agreement statistics such as intraclass correlation coefficient (ICC) and within-group interrater agreement (r-wg) could not be computed for that reason.

Two implications follow. The TOE paths test whether individually perceived organizational and environmental readiness predict individually perceived adoption readiness, which is a weaker claim than an organization-level test and should be read as such. This referent mismatch is also a plausible contributor to the organizational readiness measurement failure reported in Section 4.1, where management support and responsiveness to change are experienced very differently by staff in different roles and units; individual reports need not converge on a single coherent organizational property. Multi-informant designs that sample several respondents per organization and justify aggregation statistically are needed to test the TOE propositions at their intended level.

### 3.3 Measures

The instrument adapted TAM and TOE scales on a five-point Likert format (1 = strongly disagree to 5 = strongly agree) (Chiu et al., 2021; Jamil et al., 2025). Constructs were Technological Readiness (4 items), Organizational Readiness (4 items), Environmental Readiness (4 items; reduced to 3 after screening), Perceived Usefulness (4 items), Perceived Ease of Use (4 items; reduced to 3 after screening), AI Adoption Readiness (5 items), and HRM Effectiveness (4 items). PEOU4 (‘Internet connectivity issues do NOT hinder my use of HR tools’) was reverse-worded; preliminary screening showed that it weakened the scale, and it was removed (Weijters et al., 2013). ER3 was also removed because its exclusion raised the average variance extracted (AVE) value above 0.50. OR2 and OR4 produced negative loadings in the initial measurement model. A sensitivity analysis reversing both items raised standardized α only to 0.264 (raw-score α = 0.243), confirming that item direction alone could not restore internal consistency. Deletion decisions followed rules fixed before estimation: indicators were removed only where the outer loading fell below 0.60, and removal raised composite reliability or AVE above the recommended thresholds, and only where the construct retained at least three indicators and its conceptual domain was preserved (Hair et al., 2019). Both deletions were made during measurement assessment, before any structural path was estimated, so no indicator was removed on the basis of its effect on a hypothesis test. Two of 29 indicators were removed in total, and each removal was checked against item content: PEOU4 addressed connectivity constraints rather than ease of use as such, and ER3 addressed competitive pressure, which is weakly applicable to public-sector respondents facing no market competition.

### 3.4 Data analysis

#### 3.4.1 Quantitative analysis

PLS-SEM was estimated using the original SmartPLS 4 (SmartPLS GmbH, Bönningstedt, Germany) settings: path weighting, standardized results, a 10^−7^ stop criterion, and 10,000 bootstrap samples with two-tailed tests. Analysis proceeded in two stages: measurement assessment followed by structural testing (Hair et al., 2019). Because OR failed all reliability and convergent-validity criteria, it was excluded before the structural model was re-estimated. Significance was evaluated with t-values, *p*-values, percentile intervals, and bias-corrected and accelerated (BCa) intervals.

#### 3.4.2 Qualitative analysis

The nine transcripts were analyzed through reflexive thematic analysis (Braun and Clarke, 2008, 2019). Coding combined a deductive frame anchored in the TOE–TAM constructs with inductive codes for mechanisms not represented in the survey. Through familiarization, initial coding, and iterative theme development, five analytic themes were generated and then mapped against the quantitative results to explain convergence and divergence.

### 3.5 Common-method bias

All quantitative variables were obtained from the same respondents through a single self-administered instrument, so common-method variance is a relevant concern (Podsakoff et al., 2003). Procedural remedies were applied at the design stage. Respondents were assured of anonymity and told there were no right or wrong answers, which reduces evaluation apprehension and socially desirable responding. Predictor and criterion items were separated in the questionnaire and grouped by construct under distinct headings rather than interleaved, so that respondents were less able to infer the hypothesized relationships. Items were written in simple language, and ambiguous or double-barrelled wording was removed during piloting.

Two statistical assessments were then applied. Harman’s single-factor test, an unrotated principal components analysis of the 23 indicators retained in the final model, returned five components with eigenvalues above one, together accounting for 60.7% of the variance; the first component explained 26.50%. No single factor dominates, and the first component accounts for well under half the variance. Because Harman’s test is insensitive to moderate bias, the full collinearity procedure of Kock (2015) was also applied, in which each construct is regressed on all others in the model. The resulting variance inflation factors ranged from 1.09 to 1.69, all well below the threshold of 3.3 at which common method variance is indicated.

The observed pattern of results points the same way. Common-method variance characteristically inflates observed relationships among constructs measured by the same method. The structural results here run in the opposite direction: five of six estimable paths were non-significant, the explained variance in adoption readiness was 8.2%, and one scale failed to cohere at all. Severe method bias would have produced a uniformly well-fitting model with inflated coefficients, which is not the pattern observed. Discriminant validity was also satisfactory, with all heterotrait–monotrait (HTMT) values at or below 0.72 (Section 4.1); a single dominant method factor would be expected to push those values towards unity. Method bias cannot be excluded, and it may still affect individual coefficients, but it is unlikely to account for the study’s central findings.

### 3.6 Ethical considerations

Ethical approval was granted by the Zanzibar University Research Ethics Committee. Participants received an information sheet covering the study’s purpose, procedures, risks, and benefits, and their right to withdraw. Written informed consent was obtained before survey and interview participation, and data were anonymized using participant codes (RS1–RS9 for interviewees).

## 4 Results

### 4.1 Measurement model

Reliability and validity were assessed using criteria from Hair et al. (2019). TR, PEOU, PU, AIR, and HRME performed well: outer loadings exceeded 0.62, Cronbach’s α ranged from 0.710 to 0.884, composite reliability ranged from 0.837 to 0.904, and AVE ranged from 0.520 to 0.703. After ER3 was removed, ER also met convergent-validity requirements (AVE = 0.595). Discriminant validity was satisfactory, with all heterotrait–monotrait ratios at or below 0.72, below the 0.85 threshold (Henseler et al., 2014).

OR behaved differently. Cronbach’s α was negative (−0.105), composite reliability was 0.171, and AVE was 0.273. Its inter-item correlations ranged from −0.122 to 0.183. OR2 and OR4 loaded negatively (−0.358 and −0.217), so item direction was examined as a possible coding explanation. Reversing both increased standardized α only to 0.264 (raw-score α = 0.243), still far below accepted levels. The failure therefore extended beyond a missed reverse-coding decision. OR was excluded from structural estimation and is reported only as a measurement finding. The interview evidence in Section 4.3 is used to explore a possible institutional explanation; it does not convert the failed scale into a valid effect estimate. Three alternatives to exclusion were considered and rejected on methodological grounds. Deleting the two negatively loading items would have left a two-item scale whose remaining items correlated at *r* = 0.18, so reliability would still have failed. Respecifying the OR formatively was rejected because a formative specification must be justified conceptually before data collection rather than adopted *post hoc* to rescue a failed reflective scale, and because the indicators were written as reflective manifestations of a common construct. Retaining the four items as separate single-indicator constructs would have produced constructs whose reliability cannot be established from these data and would have multiplied tests on a sample already underpowered for small effects. Exclusion with transparent reporting was therefore preferred, and the failure is presented as a measurement finding requiring replication, not as a validated result (Table 1).

**Table 1:** Initial measurement-model results before exclusion of OR (*N* = 137).

Construct	Items retained	Loading range	α	rho_a	rho_c	AVE
AIR	AIR1–AIR5	0.633–0.777	0.770	0.783	0.844	0.520
TR	TR1–TR4	0.621–0.832	0.788	0.826	0.859	0.606
ER	ER1, ER2, ER4	0.744–0.799	0.664	0.672	0.815	0.595
PU	PU1–PU4	0.741–0.838	0.806	0.810	0.873	0.633
PEOU	PEOU1–PEOU3	0.743–0.847	0.710	0.722	0.837	0.633
HRME	HRME1–HRME4	0.760–0.927	0.884	0.868	0.904	0.703
OR	OR1–OR4	−0.358–0.730	−0.105	0.097	0.171	0.273

ER3 and PEOU4 were removed at screening. OR2 and OR4 loaded negatively. Reversing both produced a standardized *α* = 0.264 (raw-score *α* = 0.243), which remained unacceptable; OR was therefore excluded before structural testing. All HTMT values were ≤0.72.

### 4.2 Structural model

The reduced structural model excluded OR. Inner variance inflation factor (VIF) values ranged from 1.00 to 1.66, indicating no problematic collinearity. The saturated-model standardized root mean square residual (SRMR) was 0.081, while the estimated-model SRMR was 0.144. The latter exceeds the 0.08 threshold value commonly cited for approximate fit (Henseler et al., 2014) and is considered alongside the low explained variance. The model explained 30.5% of the variance in PU (*R*^2^ = 0.305), 8.2% in AIR (*R*^2^ = 0.082), and 1.0% in HRME (*R*^2^ = 0.010). PEOU → PU had a large effect (*f*^2^ = 0.438); TR → AIR remained small (*f*^2^ = 0.040). Figure 2 and Tables 2, 3 report the reduced-model results.

**Figure 2:**
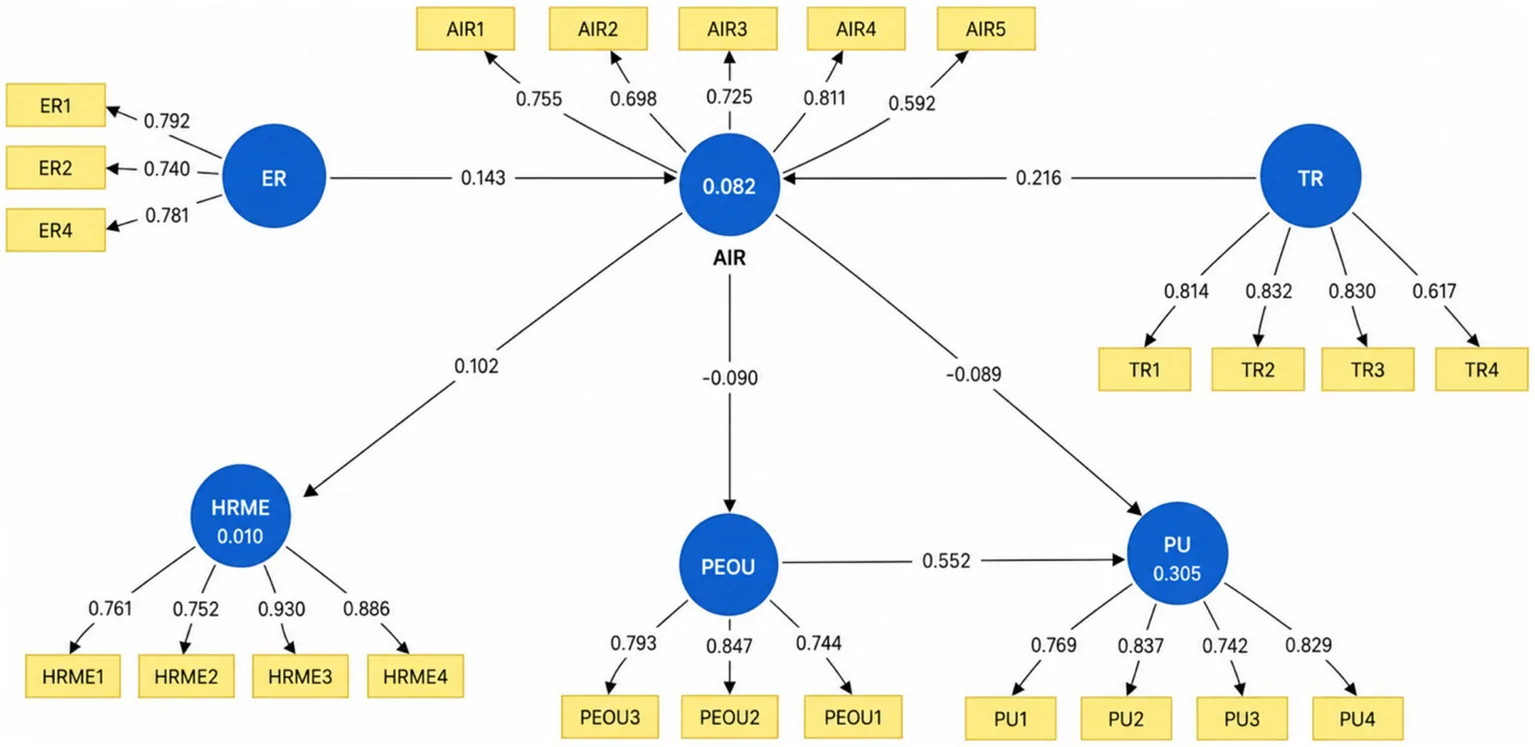
Reduced structural model after exclusion of organizational readiness. Values on arrows are standardized coefficients; values inside endogenous constructs are *R*^2^.

**Table 2:** Structurally interpretable hypothesis tests after exclusion of OR (10,000 bootstrap samples; two-tailed).

*H*	Path	*β*	*t*	*p*	95% CI (percentile)	Decision
H1	TR → AIR	0.216	2.01	0.044	[0.003, 0.406]	Marginal
H3	ER → AIR	0.143	1.17	0.243	[−0.136, 0.383]	Not supported
H4	PEOU → PU	0.552	7.67	< 0.001	[0.414, 0.692]	Supported
H5	PU → AIR	−0.089	0.83	0.407	[−0.290, 0.129]	Not supported
H6	PEOU → AIR	−0.090	0.82	0.411	[−0.316, 0.116]	Not supported
H7	AIR → HRME	0.102	0.66	0.507	[−0.245, 0.279]	Not supported

β = original-sample standardized coefficient. H1 is classified as marginal because its percentile interval narrowly excludes zero while its BCa interval [−0.096, 0.375] includes zero. H2 is omitted because OR failed measurement validity and was excluded before structural estimation. All other unsupported paths cross zero under both interval methods.

**Table 3:** Explained variance, effect sizes, collinearity, and fit for the reduced model.

Endogenous	*R*^2^ (adj.)	*f*^2^ (selected)	Fit/collinearity
AIR	0.082 (0.054)	TR → AIR 0.040; ER → AIR 0.015	SRMR (sat.) 0.081
PU	0.305 (0.299)	PEOU→PU 0.438 (large)	SRMR (est.) 0.144
HRME	0.010 (0.003)	AIR→HRME 0.011	Inner VIF 1.00–1.66

The estimated-model SRMR remains above 0.10 after OR exclusion, so neither the structural estimates nor the explained variance should be read as confirming the model; weak structural relations, omitted variables, and residual measurement limitations are all consistent with these values (see Section 4.2). Q^2^predict values, averaged over 10 repetitions of 10-fold cross-validation, were 0.165 (PU), 0.002 (AIR), and 0.012 (HRME).

Perceived Ease of Use strongly predicted PU (H4: *β* = 0.552, *t* = 7.67, *p* < 0.001). TR predicted AIR (H1: *β* = 0.216, *t* = 2.01, *p* = 0.044), but its 95% CI [0.003, 0.406] narrowly excluded zero, while the BCa interval [−0.096, 0.375] included zero. H1 therefore received marginal, method-sensitive support. ER → AIR, PU → AIR, PEOU → AIR, and AIR → HRME were non-significant. H2 was not structurally tested because OR did not meet measurement requirements.

These fit and variance results bear directly on what the model can be said to establish. SRMR is an approximate measure of the discrepancy between the empirical and model-implied correlation matrices, and it is a secondary criterion in PLS-SEM, which is variance-based and prediction-oriented rather than designed to reproduce a covariance matrix; fit indices imported from covariance-based SEM should not be treated as confirmatory evidence here (Hair et al., 2019). Read as a diagnostic rather than a verdict, the value of 0.144 indicates that the specified relations do not reproduce the observed correlation structure well. Because the retained constructs met reliability and convergent-validity requirements (Section 4.1), the misfit is attributable mainly to the structural relations rather than to the measurement of the retained constructs, whether because of paths omitted from the model, non-linearity, or unmodeled heterogeneity within the sample.

The explained variance points the same way. *R*^2^ values of 0.082 for adoption readiness and 0.010 for HRM effectiveness fall far below the levels ordinarily reported for TOE–TAM models, and they are reported here as a limitation of the specified model rather than as a substantive result in themselves. The model is accordingly not treated as validated in this setting. Three readings remain open and cannot be separated with these data: the theorized predictors may genuinely carry little weight under mandatory, resource-constrained conditions; relevant variables may be missing from the specification, which is the possibility the qualitative phase was designed to explore; and residual measurement limitations may attenuate the estimates, given that ER required item removal and OR failed outright. The study therefore presents its structural results as exploratory evidence about the limits of an imported model, and the discussion that follows is framed accordingly.

Out-of-sample predictive relevance was assessed by PLSpredict (SmartPLS GmbH, Bönningstedt, Germany) using ten-fold cross-validation, in which the model is estimated on nine folds and used to predict the indicators of the endogenous constructs in the held-out fold (Shmueli et al., 2019). Because fold assignment is random, the procedure was repeated ten times, and the results were averaged. Q^2^predict was 0.165 for perceived usefulness, 0.002 for adoption readiness, and 0.012 for HRM effectiveness; across repetitions, the adoption readiness value remained within ±0.02 of zero. Values at or near zero indicate prediction no better than the training-sample mean, so the model carries no meaningful predictive relevance for adoption readiness or HRM effectiveness, while retaining modest predictive relevance for perceived usefulness. The out-of-sample evidence therefore reproduces the in-sample pattern: the TAM mechanism linking ease of use to usefulness both holds and predicts, whereas the readiness-to-adoption and adoption-to-effectiveness relationships do neither. This strengthens rather than weakens the study’s argument, since it shows that the limited explanatory coverage is a property of the model in this setting rather than an artefact of in-sample estimation.

### 4.3 Qualitative results

Reflexive thematic analysis of the nine interviews produced five themes that contextualize the quantitative findings. Table 4 summarizes the coding frame; the themes are then presented with illustrative accounts.

**Table 4:** Coding frame derived from the interviews.

**Theme**	**Definition**	**Informants**
Stalled, partial digitalization	HR remains hybrid; only payroll and e-office are digitalized; adoption is incremental, not transformative	RS1, RS3, RS4, RS6, RS9
Infrastructure and vendor dependence as a binding constraint	Connectivity, power, devices, security, and critically, loss of control over externally owned systems	RS2, RS5, RS6, RS8, RS9
Capacity deficits and expertise flight	Skills gaps among HR users and IT staff; scarcity and loss of local AI expertise	RS1, RS3, RS5, RS6, RS7, RS8
Institutional decoupling: policy versus practice	AI as a ‘buzzword’; e-Government Authority of Zanzibar (eGAZ) as policy without operational support; public–private divide; weak political will	RS1, RS2, RS3, RS4, RS6, RS7
Cultural preference for human judgement under directive adoption	Distrust of automated decisions; status-quo bias; adoption experienced as a directive	RS1, RS2, RS3, RS5, RS7, RS9

#### 4.3.1 Theme 1: stalled, partial digitalization

Informants described HR systems that remain hybrid and only selectively digital. Adoption was characterized as incremental rather than transformative (RS1), and digitalization was described as narrow in scope:

RS6: *“The shift occurs more slowly than expected as only two systems were digitalized, these are payroll and e-office”.*

RS1: *“We have an HR management system in place; however, it is not yet fully utilized”.*

#### 4.3.2 Theme 2: infrastructure and vendor dependence as a binding constraint

Beyond connectivity, informants identified power, devices, security, and a loss of control over critical systems. The sovereignty problem was stated directly:

RS6: *“Still infrastructure is a major impediment to AI-HR as till now the government depends on private sector in digital systems. For example, the current server is not owned by the government, but by a private company headquartered in Tanga”.*

RS2: *“Vendor Dependence and Limited Customization: Heavy reliance on external vendors, with limited access to source code or configuration flexibility, restricts system adaptation to local policies and evolving HR needs”.*

RS9: *“Lack of good IT infrastructure some parts in Zanzibar have no internet connection or limited connection up to 3G or 2G … lack of modern computer devices; most use old versions which may not run AI tools”.*

#### 4.3.3 Theme 3: capacity deficits and expertise flight

Skills gaps were located primarily among HR users, but also among IT staff, and were compounded by the loss of scarce local expertise:

RS1: *“The skills gap exists on both sides, but it is more pronounced among HR staff who use the systems daily … which leads to underutilization of existing systems”.*

RS7: *“In the government also they lack expertise in AI. In the private sector too … we have more than 200 employees but very few … understand AI”.*

RS5: *“There was one staff member at eGAZ who was very good in designing system but the government no longer uses him”.*

#### 4.3.4 Theme 4: institutional decoupling: policy versus practice

Informants consistently described a gap between formal policy and operational reality. AI was widely treated as a “buzzword,” and government initiatives were experienced as documents rather than support:

RS7: *“Much of the support remains at the policy and strategic level, with limited practical implementation … there is still a gap between the stated objectives and real, on-the-ground digital transformation outcomes”.*

RS6: *“Just policy documents no any change I have seen from these initiatives”.*

RS3: *“Still AI in HR is buzzword, it is designed to satisfy organization in Global North and not in Zanzibar”.*

The decoupling had a distinct public–private dimension and a political-will dimension:

RS7: *“All investment in AI is posed in the government sectors and nothing done in private sectors”.*

RS9: *“Limited political will; most of employees in Zanzibar usually implement what has been directed by elected politicians. So when they do not emphasize use of this technology it cannot be adopted”.*

#### 4.3.5 Theme 5: cultural preference for human judgment under directive adoption

Informants reported low trust in automated decisions and a normative attachment to human judgment, alongside adoption experienced as directive rather than chosen:

RS1: *“Employees generally prefer the human touch … trust in purely algorithmic decisions is low, and people often expect a human to review or validate outcomes”.*

RS3: *“HR could not be integrated by AI; our HR system depend much on rules, regulations, Act and policies. These all depend on human touch, human thinking, discussion and group decisions other than automation”.*

RS5: *“Many teachers resist to use digital system for examination, results and biometric machine. They believe manual system is better … But I believe when more promotion [is] available people will change their mindsets”.*

## 5 Discussion

### 5.1 Overall pattern: interpreting the unexplained variance

The reduced model explained 8.2% of the variance in adoption readiness and 1.0% in HRM effectiveness. These low values indicate that the specified TOE–TAM variables provide limited explanatory coverage in this sample. The interviews offer plausible contextual mechanisms for the unexplained variance, but omitted variables and measurement limitations may also contribute; ER required item removal, and OR failed. The qualitative phase therefore supports explanation and theory development without treating every null result as proof of institutional decoupling. Figure 3 summarizes the integration, and Table 5 links each unsupported relationship or measurement finding to the interview evidence used in the interpretation. Integration occurred at three points and is assessed against the criteria for mixed-methods integration set out by Fetters et al. (2013). At the design level, the explanatory sequential structure made the quantitative results the focus of what the qualitative phase had to account for. At the methods level, informants were selected for their capacity to speak to the specific non-significant paths, and the interview guide was organized around the TOE–TAM constructs so that themes could be mapped onto hypotheses. At the interpretation and reporting level, integration is presented through the joint display (Figure 3) and the path-by-path mapping (Table 5).

**Figure 3:**
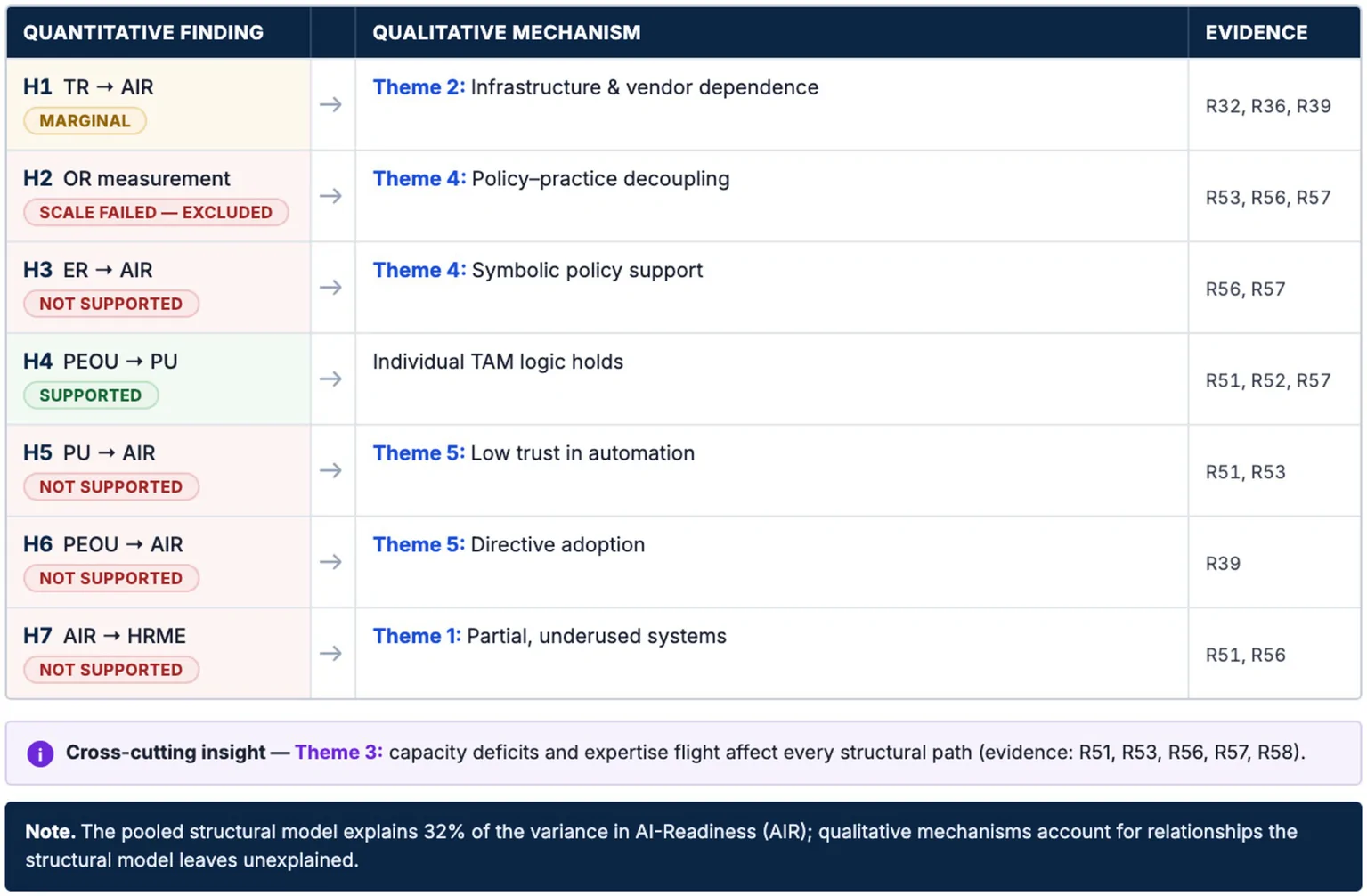
Joint display integrating the reduced quantitative model and the qualitative mechanisms used to interpret its results (*N* = 137; nine key-informant interviews, RS1–RS9).

**Table 5:** Explicit qualitative–quantitative mapping for unsupported paths and the OR measurement finding.

**Finding**	**Theme**	**Representative interview evidence**	**Explanatory link**
OR measurement failure	Theme 4: institutional decoupling	RS6: ‘Just policy documents no any change I have seen from these initiatives’.	Formal support and operational responsiveness may not move together; OR was not tested structurally.
H3: ER → AIR	Theme 4: policy versus practice	RS7: ‘Much of the support remains at the policy and strategic level, with limited practical implementation’.	Policy visibility may not transmit resources or implementation support to organizations.
H5: PU → AIR	Theme 5: human judgement	RS1: ‘trust in purely algorithmic decisions is low’.	Perceived value may not produce readiness when automated decisions lack legitimacy.
H6: PEOU → AIR	Theme 5: directive adoption	RS9: employees ‘implement what has been directed by elected politicians’.	Ease of use cannot replace authorization or implementation support in a directive setting.
H7: AIR → HRME	Theme 1: partial digitalization	RS1: ‘We have an HR management system in place; however, it is not yet fully utilized’.	Readiness without routinized use may not yield observable effectiveness gains.

Quotations are shortened only for display; the fuller excerpts appear in Section 4.3. The interview evidence provides plausible explanations and is not treated as a causal test of the null paths.

Assessing the fit between the two strands yields three kinds of relationship rather than uniform agreement, and stating them separately guards against reading the qualitative material as a confirmation of the quantitative results. The strands confirm one another for H4 and, more weakly, H1: informants described usability as a real but secondary concern relative to infrastructure, which is consistent with a strong PEOU → PU path alongside a marginal TR → AIR path. They expand one another for the null paths and the OR failure, where the interviews supply mechanisms the survey did not measure, such as vendor dependence, expertise flight, and directive adoption; expansion is the dominant form of integration in this study and is what the low explained variance would lead one to expect. There is also one point of discordance that should not be smoothed over: informants reported concrete operational gains where systems are embedded, including improved data accessibility and faster document drafting, whereas the AIR → HRME path was null. The most plausible reconciliation is that benefits accrue within specific routinized functions rather than as the organization-wide effectiveness that the HRME scale measures, but the design cannot adjudicate this, and it is carried forward as a research question in Section 6.3.

The inferences drawn from this integration are explanatory rather than confirmatory. Qualitative accounts can identify mechanisms that a survey omitted and can make a null result intelligible, but they cannot establish that those mechanisms produced the null result, and nine informants cannot establish their prevalence across the 137 surveyed respondents. The mappings in Table 5 are therefore proposed as candidate explanations to be tested, principally by measuring the identified mechanisms directly in subsequent quantitative work.

### 5.2 Hypothesis-by-hypothesis analysis

#### 5.2.1 H1: technological readiness → adoption readiness (marginal)

TR was the largest predictor of AIR in the reduced model (β = 0.216, *p* = 0.044), but the effect was small (f^2^ = 0.040) and method-sensitive because the BCa interval included zero. It should not be treated as a robust independent predictor. Theme 2 specifies what technological readiness entails locally: connectivity, power, devices, security, and control over systems (RS2, RS6, RS9). These accounts support a tentative threshold interpretation that requires testing in larger or longitudinal samples.

#### 5.2.2 Organizational readiness: measurement failure (H2 not structurally tested)

OR failed measurement validity and was excluded before the structural model was estimated, so no OR → AIR coefficient is reported or interpreted. The result is confined to measurement: management support, resources, and responsiveness to change did not constitute a coherent reflective construct in this sample. Theme 4 offers one possible explanation. RS6 described government initiatives as ‘just policy documents’, while RS3 called AI in HR a ‘buzzword’. These accounts are consistent with institutional decoupling (Boxenbaum and Jonsson, 2008), but the interview evidence is interpretive and does not validate the failed OR scale.

#### 5.2.3 H3: environmental readiness → adoption readiness (not supported)

ER did not predict AIR in the reduced model (*β* = 0.143, *p* = 0.243). Theme 4 provides the explanatory link: RS7 described support as remaining at the policy and strategic level, with little practical implementation, while RS6 reported seeing no change beyond policy documents. The null path may therefore reflect limited resource transmission from policy to organizations. It may also reflect measurement limitations, because ER3 was removed during screening.

#### 5.2.4 H4: perceived ease of use → perceived usefulness (supported)

PEOU strongly predicted PU (β = 0.552, *p* < 0.001), confirming that the individual cognitive evaluation of technology operated as theorized. Informants treated usability and English–Swahili interface issues as real but secondary to infrastructure and governance constraints (RS1, RS2, RS7). The TAM relationship held at the individual level, although it did not extend to organizational adoption readiness.

#### 5.2.5 H5: perceived usefulness → adoption readiness (not supported)

PU did not predict AIR (*β* = −0.089, *p* = 0.407). Theme 5 helps explain why perceived value may not translate into organizational readiness. RS1 reported low trust in purely algorithmic decisions, and RS3 stressed continued dependence on human discussion and judgement. Perceived usefulness alone may therefore be insufficient where automated decisions lack legitimacy, and HR professionals do not control adoption.

#### 5.2.6 H6: perceived ease of use → adoption readiness (not supported)

PEOU did not predict AIR (β = −0.090, *p* = 0.411). RS9’s account provides the clearest explanatory link: employees implement technology when elected leaders direct them to do so. Under directive adoption, ease of use cannot substitute for authorization, infrastructure, or implementation support. The result suggests that voluntariness should be tested as a moderator rather than assumed.

#### 5.2.7 H7: adoption readiness → HRM effectiveness (not supported)

AIR did not predict HRME (β = 0.102, *p* = 0.507). Theme 1 provides a direct explanation: RS1 reported that the existing HR system was not yet fully utilized, and RS6 described digitalization as limited to payroll and e-office. Readiness without routinized use may not generate observable performance gains. A cross-sectional design cannot establish whether such gains emerge later, so the temporal-lag interpretation remains provisional.

### 5.3 Toward a context-sensitive account of adoption: five testable propositions

The Zanzibar evidence motivates five propositions about how TOE–TAM may behave in resource-constrained small island contexts. They are advanced as hypotheses for testing rather than as established refinements of the framework, because they rest on one underpowered cross-sectional sample, a model with low explained variance, and interview accounts that identify mechanisms without establishing them. First, technological readiness may function as a threshold condition rather than a continuous predictor. This proposition is the most tentative of the five: H1 was marginal and method-sensitive, its effect size was small, and the threshold interpretation comes from the interviews rather than from a tested discontinuity, which would require a model specified to detect one. Second, external vendor control may restrict organizational agency in ways that TOE, as conventionally specified, does not capture, which suggests treating technology sovereignty as a candidate construct; it was not measured in this study, and the proposition rests on interview evidence alone. Third, mandatory adoption may weaken the link between individual perceptions and organizational readiness. Voluntariness was not measured or manipulated here, so this remains an interpretation of null paths and requires a design in which it is tested as a moderator. Fourth, formal policy may remain disconnected from operational practice. The OR measurement failure is consistent with this reading and indicates that imported readiness scales may not travel intact, although a failed scale is evidence about measurement rather than a demonstration of decoupling. Fifth, the readiness–effectiveness relationship may be temporally lagged; a cross-sectional null result is equally consistent with no relationship, so only longitudinal evidence can distinguish the two. These propositions are context-derived. They should not be generalized to other small island or low-resource settings without comparative evidence, and they should not be treated as a validated contextual variant of TOE–TAM until they are tested in samples powered to detect the effects concerned (Table 6).

**Table 6:** TOE–TAM expectations and the Zanzibar findings, with the evidential status of each contrast.

Factor	Resource-rich benchmark settings	Zanzibar (small island context)
Infrastructure	Assumed reliable	Possible threshold condition (H1 marginal)
Organizational readiness	Operationally meaningful	Scale failed to cohere; decoupling is one interpretation
Environmental readiness	Policy drives behavior	No association observed; symbolic compliance is a candidate explanation
TAM (PU/PEOU)	Predicts adoption	Perception–adoption link not observed; voluntariness untested
Adoption → effectiveness	Contemporaneous	No association observed; lag untested
Vendor sovereignty	Assumed internal	Externally controlled (interview evidence; not measured)

## 6 Conclusion

This study examined AI-enabled HR adoption in Zanzibar’s public and private sectors through an explanatory sequential design. After the invalid OR measurement was removed, technological readiness was the largest predictor of adoption readiness, though only marginally and in a method-sensitive manner; with the sample powered to detect standardized effects of about 0.21 or larger, this association should be regarded as provisional. ER, PU, and PEOU did not predict AIR, while AIR did not predict HRME. Nine interviews supplied five candidate mechanisms that the model did not measure: stalled digitalization, infrastructure and vendor dependence, capacity and expertise deficits, institutional decoupling, and a cultural preference for human judgement under directive adoption.

### 6.1 Practical implications

Policy should connect digital-HR strategies to budgets, training, implementation responsibility, and monitoring. The small and method-sensitive TR association does not, by itself, justify a large infrastructure program; the recommendation is supported jointly by the quantitative direction and repeated interview evidence on connectivity, power, devices, security, and externally controlled servers. Capacity building should provide credentialed, bilingual training for HR users and IT staff and retain scarce local expertise. A phased strategy can begin with lower-sensitivity functions such as training and recruitment before moving to payroll and contracts, and evaluation should track actual use and HR outcomes rather than formal readiness alone.

### 6.2 Limitations

The design is cross-sectional, which constrains the analysis in three ways. Causal direction cannot be established for any estimated path, so relationships that are theorized as causal are tested only as associations, and reverse or reciprocal effects, such as effective HR practice raising perceived readiness, remain equally consistent with the data. Effects that require time to materialize cannot be detected, so the non-significant H7 path may reflect a lag rather than an absence of effect. Because predictors and outcomes were measured at the same moment from the same respondents, temporal separation was unavailable as a remedy for method bias. AIR R^2^ was only 0.082, and the estimated-model SRMR remained high (0.144); omitted variables and measurement limitations are therefore credible alternatives to the contextual explanation developed from the interviews. ER required item removal, and OR failed. Future studies should develop context-specific, multidimensional organizational-capacity measures rather than importing a reflective scale without local validation. All quantitative data were self-reported and collected through a single instrument. Procedural remedies, Harman’s single-factor test, and a full collinearity assessment are reported in Section 3.6 and indicate no evidence of substantial method bias, but the risk cannot be eliminated, and marker-variable or multi-source designs would provide stronger protection. System logs and implementation records would allow adoption and effectiveness to be measured independently of respondent perception. Generalization is limited in three respects. The sample supports analytic rather than statistical generalization: it was drawn without a sampling frame, is weighted towards the public sector, and contains few SMEs and IT specialists (Section 3.2.1), so the results characterize public and large formal organizations more accurately than the wider population of Zanzibari employers. It is also underpowered for standardized effects below approximately 0.21, so several null paths are statistically inconclusive rather than demonstrations of absence. The nine qualitative informants were selected for information power and cannot represent all sectors. Findings from a single semi-autonomous jurisdiction should be treated as transferable to settings with comparable infrastructure, governance, and resource conditions only where that comparability is established, and not as generalizable to small island states as a class. AI-HR was nascent at data collection, making replication as implementation matures important.

### 6.3 Future research

Longitudinal designs should track organizations from installation through routinization and value realization. Comparative studies across officially designated SIDS, African island jurisdictions, and mainland settings could test whether the proposed threshold and decoupling mechanisms travel beyond Zanzibar. Studies comparing mandatory and voluntary adoption could clarify when TAM’s attitude-to-adoption link weakens. Intervention research on infrastructure and locally controlled systems would provide stronger causal evidence. Future measurement studies should also develop and validate a sociocultural-readiness construct covering preferences for human judgement, data-sovereignty concerns, and expertise-retention vulnerability.

In Zanzibar’s resource-constrained public and private sectors, the conventional readiness and acceptance predictors explained little of the variation in AI-HR adoption, while informants pointed consistently to infrastructure, institutional coherence, technology sovereignty, and sociocultural fit. The study therefore contributes a documented case in which an imported adoption model underperforms, together with a set of testable propositions about why, rather than a validated contextual theory. Its practical value lies in directing attention and evaluation effort to those conditions.

## Data Availability

The original contributions presented in the study are included in the article/Supplementary material, further inquiries can be directed to the corresponding author/s.
