# Supplementary material for: When readiness fails: a TOE–TAM mixed-methods analysis of AI-enabled HR digitalization in a resource-constrained small island context
Source: Front Artif Intell. 2026 Sep 21;9:1902314. doi: 10.3389/frai.2026.1902314 (PMC13635292; doi:10.3389/frai.2026.1902314)
Supplement: Supplementary file 1 [file Supplementary_file_1.DOCX]

**Survey: AI-Enabled HR Adoption in Zanzibar**

**Purpose:** This survey aims to understand the readiness and adoption of AI and digital tools in Human Resource (HR) processes in Zanzibar. Your responses are confidential and will be used for academic research only.

**Instructions:** Please answer all questions honestly. There are no right or wrong answers.

**Section A: Demographic Profile**

*(Used for group analysis, e.g., Public vs. Private sector)*

1. **Type of Organization:**
   [ ] Public Sector / Government Agency
   [ ] Private Sector
   [ ] SME (Small/Medium Enterprise)
   [ ] NGO / Civil Society
2. **Your Job Role:**
   [ ] HR Manager/Staff
   [ ] IT Manager/Staff
   [ ] General Management
   [ ] Other
3. **Years of Experience:**
   [ ] Less than 1 year
   [ ] 1–5 years
   [ ] 6–10 years
   [ ] Over 10 years

**Section B: AI-Enabled HR Adoption (AIR)**

*Please indicate the level of automation for the following HR functions in your organization.*
*(Scale: 1 = Manual/Paper-based, 2 = Basic IT (Excel), 3 = Specialized Software, 4 = Fully Automated/AI)*

| **Variable: AI Adoption (AIR)** | **Manual (1)** | **Basic IT (2)** | **Software (3)** | **AI/Auto (4)** |
| --- | --- | --- | --- | --- |
| **AIR1.** Recruitment & Selection | [ ] | [ ] | [ ] | [ ] |
| **AIR2.** Payroll Processing | [ ] | [ ] | [ ] | [ ] |
| **AIR3.** Performance Appraisal | [ ] | [ ] | [ ] | [ ] |
| **AIR4.** Employee Records Management | [ ] | [ ] | [ ] | [ ] |
| **AIR5.** Training & Development | [ ] | [ ] | [ ] | [ ] |

**Section C: Technology-Organization-Environment (TOE)**

*To what extent do you agree with the following statements?*
*(Scale: 1 = Strongly Disagree to 5 = Strongly Agree)*

| **Technological Readiness (TR)** | **1** | **2** | **3** | **4** | **5** |
| --- | --- | --- | --- | --- | --- |
| **TR1.** Our organization has reliable internet connectivity for cloud HR tools. | [] | [] | [] | [] | [] |
| **TR2.** Our current hardware/software is compatible with modern AI tools. | [] | [] | [] | [] | [] |
| **TR3.** We have robust data security measures to protect employee privacy. | [] | [] | [] | [] | [] |
| **TR4.** HR data in our organization is standardized and easy to access. | [] | [] | [] | [] | [] |
| **Organizational Readiness (OR)** | **1** | **2** | **3** | **4** | **5** |
| **OR1.** Top management actively supports the shift to digital HR. | [] | [] | [] | [] | [ |
| **OR2.** Our HR staff possesses the necessary technical skills for AI tools. | [] | [] | [] | [] | [] |
| **OR3.** The organization has allocated sufficient budget for digital transformation. | [] | [] | [] | [] | [] |
| **OR4.** Employees are open to changing from paper-based to digital workflows. | [] | [] | [] | [] | [] |
| **Environmental Readiness (ER)** | **1** | **2** | **3** | **4** | **5** |
| **ER1.** Government initiatives (e.g., eGAZ) encourage us to adopt digital HR. | [] | [] | [] | [] | [] |
| **ER2.** Current labor laws/policies support the use of AI in HR. | [] | [] | [] | [] | [] |
| **ER3.** We face pressure from competitors/partners to modernize our HR systems. | [] | [] | [] | [] | [] |
| **ER4.** National culture supports the use of automated decision-making. | [] | [] | [] | [ ] | [ ] |

**Section D: Technology Acceptance (TAM)**

*How do you perceive the value of AI tools in HR?*
*(Scale: 1 = Strongly Disagree to 5 = Strongly Agree)*

| **Perceived Usefulness (PU)** | **1** | **2** | **3** | **4** | **5** |
| --- | --- | --- | --- | --- | --- |
| **PU1.** Using AI-enabled tools would speed up our HR processes. | [ ] | [ ] | [ ] | [ ] | [ ] |
| **PU2.** AI would increase fairness/objectivity in recruitment & reviews. | [ ] | [ ] | [ ] | [ ] | [ ] |
| **PU3.** Digital HR systems would make my job easier to perform. | [ ] | [ ] | [ ] | [ ] | [ ] |
| **PU4.** AI tools would improve the quality of our HR decision-making. | [ ] | [ ] | [ ] | [ ] | [ ] |
| **Perceived Ease of Use (PEOU)** | **1** | **2** | **3** | **4** | **5** |
| **PEOU1.** Learning to operate new HR software is easy for me. | [ ] | [ ] | [ ] | [ ] | [ ] |
| **PEOU2.** I find it easy to get digital HR systems to do what I want. | [ ] | [ ] | [ ] | [ ] | [ ] |
| **PEOU3.** Overall, I find AI-enabled HR tools easy to understand and use. | [ ] | [ ] | [ ] | [ ] | [ ] |
| **PEOU4.** Internet connectivity issues do NOT hinder my use of HR tools. | [ ] | [ ] | [ ] | [ ] | [ ] |

*(Note: PEOU4 is phrased positively here to avoid confusion in analysis)*

**Section E: HRM Effectiveness (HRME)**

*How would you rate the current performance of the HR function in your organization?*
*(Scale: 1 = Very Poor, 2 = Poor, 3 = Average, 4 = Good, 5 = Excellent)*

| **HRM Effectiveness (HRME)** | **1** | **2** | **3** | **4** | **5** |
| --- | --- | --- | --- | --- | --- |
| **HRME1.** Speed of resolving employee requests (e.g., leave, payroll). | [ ] | [ ] | [ ] | [ ] | [ ] |
| **HRME2.** Accuracy of HR data and reports. | [ ] | [ ] | [ ] | [ ] | [ ] |
| **HRME3.** Fairness and transparency in recruitment and appraisals. | [ ] | [ ] | [ ] | [ ] | [ ] |
| **HRME4.** Ability to attract and retain high-performing talent. | [ ] | [ ] | [ ] | [ ] | [ ] |
